# Smart Meeting Room Usage Information and Prediction by Modelling Occupancy Profiles †

**DOI:** 10.3390/s19020353

**Published:** 2019-01-16

**Authors:** Unai Saralegui, Miguel Ángel Antón, Olatz Arbelaitz, Javier Muguerza

**Affiliations:** 1 Tecnalia Research & Innovation, Mikeletegi 2, 20009 Donostia, Spain; mangel.anton@tecnalia.com; 2 Faculty of Informatics, University of the Basque Country, UPV/EHU, 20018 Donostia, Spain; olatz.arbelaitz@ehu.eus (O.A.); j.muguerza@ehu.eus (J.M.)

**Keywords:** buildings, ambient intelligence, occupancy detection, behaviour modelling, sensor networks, smart meeting room, Internet of Things (IoT)

## Abstract

The monitoring of small houses and rooms has become possible due to the advances in IoT sensors, actuators and low power communication protocols in the last few years. As buildings are one of the biggest energy consuming entities, monitoring them has great interest for trying to avoid non-necessary energy waste. Moreover, human behaviour has been reported as being the main discrepancy source between energy usage simulations and real usage, so the ability to monitor and predict actions as opening windows, using rooms, etc. is gaining attention to develop stronger models which may lead to reduce the overall energy consumption of buildings, considering buildings thermal inertia and additional capabilities. In this paper, a case study is described in which four meeting rooms have been monitored to obtain information about the usage of the rooms and later use it to predict their future usage. The results show the possibility to deploy a simple and non-intrusive sensing system whose output could be used to develop advanced control strategies.

## 1. Introduction

Internet of Things (IoT) devices are becoming popular both for sensing and actuating. The integration of those systems is rapidly increasing, and it is expected to continue to do so; different entities have forecasted different increase levels for the next years, but most estimations forecast between 20 to 30 billion connected devices by 2020 [1]. Several fields have evolved influenced by the advances in the IoT paradigm; the way these devices offer simple and affordable monitoring and actuating functionalities has facilitated the development of various research paths in the past years.

One of the areas that has received a great impulse from the IoT field advances is the smart building framework. This area is gaining increasing attention as people spend most part of their time inside a building, including their home, office, restaurant, etc. Considering that, it is not a surprise that buildings are responsible for about 40% of the energy consumption in developed countries, accounting for a larger proportion of the total energy consumption than both the industry and transportation [2]. Moreover, such energy usage is expected to grow about 45% over the next 20 years [3], thus gaining knowledge about the energy usage and finding ways to reduce such consumption is a common goal sought by researchers and practitioners.

On most actions related with the energy consumption in buildings the user is direct or indirectly involved, whether it is switching on/off lights, adjusting the air conditioning temperature, etc. A proper modelling and prediction of those actions could greatly reduce the energy consumption. That is why developing sensing networks and predictive systems is gaining increasing attention in the field. One of the methodologies to deal with predictive systems is the Model Predictive Control (MPC), which considers real building models (designed for example with EnergyPlus), and predictive models to develop control strategies mainly for heating, ventilating and air-conditioning (HVAC) systems.

In this paper, the presented solution is proved in four smart meeting rooms (SMR), which are a sub-discipline of Ambient-Intelligent environments [4]. SMR are referred as intelligent systems that are able to extract information from past events and reuse such knowledge to detect behaviour patterns and predict similar behaviours in the future [5,6]. The approach presented in this paper consists of monitoring room level usage, and a posterior prediction of room usage using pre-calculated usage patterns. Such information is valid on its own, but it could be used as an input for MPC systems to correct the HVAC control strategies for example. The obtained usage information has also been used to contrast real usage with the room reservations registered in the booking system.

We present a valid solution to deploy a sensing network to gather data in four meeting rooms of an office-building of a Technology Centre placed in Derio (Spain), located in the Bay of Biscay in a region whose climate is classified as oceanic. The presented network is wirelessly interconnected making it possible to gain valuable knowledge without making much changes in the environment. The knowledge obtained by performing a data analysis is shown, by presenting the usage patterns of each monitored room and validating the usage patterns as predictive models. Finally, we discuss on the results obtained and on the application of the proposed methodology to make corrections in MPC strategies.

The article has the following structure: in Section 2 the work related to this paper is presented; later in Section 3 the material and techniques used to develop this research are introduced. After that, in Section 4 the obtained results are presented. Finally, the results obtained during the study are discussed and future research paths are described in Section 5.

## 2. Related Work

Recent advances in IoT, big data and machine learning algorithms have driven advances in many fields including smart buildings. The smartization of buildings requires to gather data from various sources, which requires to place a considerable number of sensors making as few changes as possible and covering various assets in the building. The ability to communicate via various wireless protocols and to develop scalable architectures to easily include new sensors make IoT devices the way to go in building monitoring. This kind of systems is used in buildings for monitoring various behaviours, usually comfort and energy usage related parameters [7]. The multiple possible inputs and architecture options create a wide variety of configuration possibilities. Those systems which allow scalability are the ones which have a brighter future as they allow to easily deploy new nodes in the installation [8].

In the actual digitisation era there is great interest in monitoring as much assets as possible, with policies for doing so being established in many developed countries as in South Korea, where BEMS installation was made mandatory for public buildings over 10,000 m${}^{2}$ [9]. According to smart homes several efforts have been made to develop energy management systems, as in [10] where they introduce a home energy management system (HEMS) to collect status and power consumption demand from home appliances. Research has also been focused in the field of machine-to-machine communications as in [11,12] where they make use of the ZigBee communication protocol for developing a HEMS.

As previously mentioned, the interaction of users with the built environment affects greatly the energy usage, being the main reason of the differences between energy usage simulations and real usage [13,14]. Due to that fact, several efforts are being made to model human behaviour in indoor spaces. According to human-building interaction, various research paths are opened for both detection and prediction [13]. This area covers research in occupancy detection (binary), occupancy estimation (amount of people in a room), window opening/closing prediction, etc. According to the occupancy detection and estimation various solutions have been proposed as it is not a directly measurable quantity, from PIR (Passive Infrared) motion detectors and CO${}_{2}$ sensors to smart metering data [15]. Those indirect ways are primarily used instead of cameras as they are not intrusive for people, even if the accuracy obtained with images from video recordings is usually higher and are commonly used for setting the ground truth in test scenarios [16].

Occupancy detection and estimation by using PIR sensors receives great attention as they are easily installable and provide a non-intrusive measuring method in contrast with regularly used cameras, which may suffer from cyberattacks and non-desired access to the recordings [17]. PIR sensors have been applied to this problem in multiple ways: a unique PIR sensor, multiple PIR sensors working together [18,19] or merged with data from other sensors such as temperature, humidity, etc. [14,20]. The application of machine learning methods has also been studied to infer occupancy and activity patterns from various data sources directly or indirectly related with human presence [21]. Recently other possibilities have been studied to detect presence by using wireless sensing possibilities provided by Wi-Fi and Bluetooth signals [22,23,24], and by monitoring personal computer usage by detecting keyboard and mouse movements [25].

The knowledge acquired by analysing the interaction between users and the building itself may be used to develop advanced control strategies where not only online measures can be used but also predictions are used to improve the control, this is the idea behind the MPC [26]. This strategy considers the actions that a user can perform to try to predict them in advance to make the necessary changes before the user acts from an energy saving perspective [27]. To be able to develop valid MPC strategies it is necessary to process the information in real time to obtain real values from the building and to make predictions in short and long term to forecast user behaviours, requiring to deploy a sensing network able to provide the necessary information in real time [28]. The required computing power to handle these scenarios may benefit from developments in cloud computing services as in [29] where they propose a building energy management system that takes advantage of available cloud services to reduce cost and installed equipment.

The room usage information is not only applicable to develop advanced control strategies, but they have also been applied to check real usage with room booking systems [17], especially in office buildings in which the private space is frequently needed and having them blocked and unused results in unnecessary wastes [30]. In addition, the wastes are not only economic as Corporate Real Estate (CRE) decisions address challenges such as productivity, employees’ well-being, innovation and flexibility; for which pervasive technologies offer potential for increasing workplace efficiency on a long-term basis [31].

## 3. Materials and Methods

**Figure 1:**
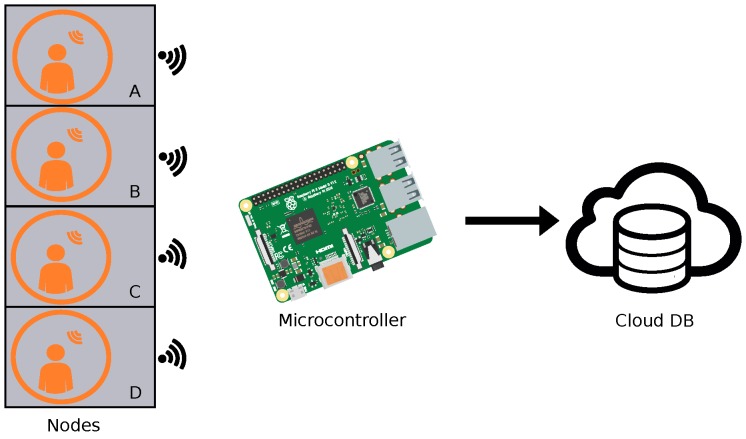
Configuration of the deployed IoT sensing equipment.

One of the main advantages of the development of wireless IoT equipment is that there is no need for a wired installation between devices, that way the effort when deploying such a network is considerably reduced. The solution proposed in this paper consists of IoT sensors as nodes (view Figure 1), with at least one node connected per monitored meeting-room, and a microcontroller responsible for receiving the data from the nodes, store the data in a database and making it accessible via cloud.

The use of wireless IoT nodes benefits the system we propose in the way that it allows to reduce the changes to be made in the rooms to be monitored, allowing for fast installations in cases in which many rooms in a building have to be equipped with the proposed configuration [32], this way the proposed system gains in scalability. Not only does the wireless nature of the systems benefit the installation, but it will also facilitate the relocation and re-usability of the sensors in other places at a very low cost in time and money. This way the proposed system is scalable and reusable with the counterpart of having to replace batteries from time to time, which should last at least for two years with the adequate configuration and taking advantage of low power communication protocols.

The proposed configuration was tested in an office building of a Research and Development centre, equipped with advanced HVAC systems. Four meeting rooms of the mentioned building where equipped with sensors to monitor presence and thermal behaviour related data. The monitored rooms are mainly used for meetings, both physical and virtual, and are reserved using the intranet of the company. In this test case the Multisensor 6 by Aeotec, which contains six built-in sensors: temperature, relative humidity, ultra-violet radiation, vibration, light and motion, were used. Such multisensor device has built-in Z-Wave low power communication, which was used to establish the communication between the sensing devices and the main node. This communication protocol is mainly used in home automation applications, it makes use of low energy radio signals for communication between devices allowing wireless control of residential appliances. Moreover, the protocol provides interoperability between home control systems of different manufacturers. The previously mentioned wireless nodes have enough smartness to send the data when there is a change in the measured value, this way the power consumption is low, which is necessary as the nodes are powered by batteries while the database does not increase with redundant data.

Our aim here is to develop an easily installable and portable system which offers facilities to move between different spaces while maintaining its functionalities, consequently other variables, a priori more related to human presence such as CO${}_{2}$ or sound, have not been considered here. CO${}_{2}$ and sound measurements suffer from great changes in small and middle sized rooms due to the nearness of occupants, that way the behaviour of the system could arise false positives or negatives once the behaviour of the system is established but its location is changed for whatever reason. This argument could also be applied for PIR movement sensors, but due to their lower price it is possible to deploy more than one sensor per room, constructing a virtual movement sensor whose output is the result of using a logical OR operation of all PIR sensor outputs, to avoid location problems while maintaining a low cost.

The main node in the test scenario consisted of a Raspberry Pi 3 model B which was equipped with a RaZberry card to communicate via the Z-Wave protocol with the sensing devices. The system runs the GNU/Linux-based Raspbian 8.0 distribution. As commented before, the main role of this node is to receive the data from all the rooms via wireless communication and store it. The received data is locally stored in a typical SQLite database, data can later be accessed from outside via RESTful web services.

As previously mentioned the installed multisensors measure motion which is in fact a binary value representing movement or lack of it. Consequently, they do not directly give presence information, so a modelling needs to be performed to obtain it [27]. In this case an automatic data modelling process has been implemented to convert motion data from five-minute interval to binary occupancy data; motion data obtained in every five-minute period is considered to determine the occupancy of the room in those five minutes, the pseudo-code of the modelling procedure is shown in Algorithm 1.
**Algorithm 1** Algorithm to model 30 minute occupancy level
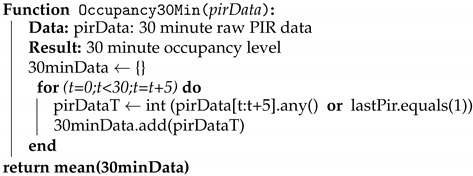


After the first modelling step in which five-minute binary occupancy data is obtained, the data goes through a second step in which such five-minute information is averaged in thirty minute bins to obtain a more representative value which gives an idea of the room usage, this is done to avoid sensor misreading when there is no much movement, for example when meeting attendants are seated and no movement is detected by the sensor [33].

**Figure 2:**
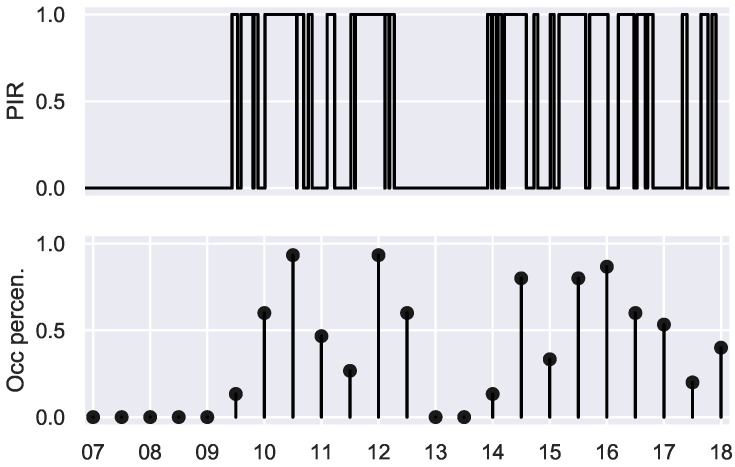
Modelling performed to obtain a percentage of presence from the raw PIR data for a workday.

The patterns shown in Figure 2 give an idea of the room usage as a percentage in a regular basis, that way, they do not directly tell if a room is used or not but give a clear idea of how the rooms are regularly used, which can be used to determine day periods when the rooms are most likely to be occupied. In certain applications like HVAC systems controlling; having this knowledge may lead to develop advanced control strategies which could in fact reduce considerably the amount of energy used in non-necessary time periods.

The pattern in Figure 2 corresponds to a day with a working time table from 8:30 to 17:30. It can be seen that there is a clear descent in use from 13:00 to 14:00 which corresponds with lunch time in the building. The pattern shows high usage in the morning and in the afternoon in this particular case, giving an idea of how the already explained data modelling works.

## 4. Results and Discussion

As mentioned in Section 3, the installed multisensors have six built-in sensors whose data was analysed for the monitoring period which lasted from July to December, that way capturing data from the two working time-tables (the first one is valid from June to mid-September and the second one is valid for the rest of the year) followed by the occupants of the building. In this case due to the artificial control of temperature and humidity performed by the HVAC systems installed in the building, it is not expected to suffer from big changes in temperature nor humidity by external factors. Due to that behaviour, it was not possible to detect significant variances in temperature and humidity, so presence related information could not be obtained from them.

**Figure 3:**
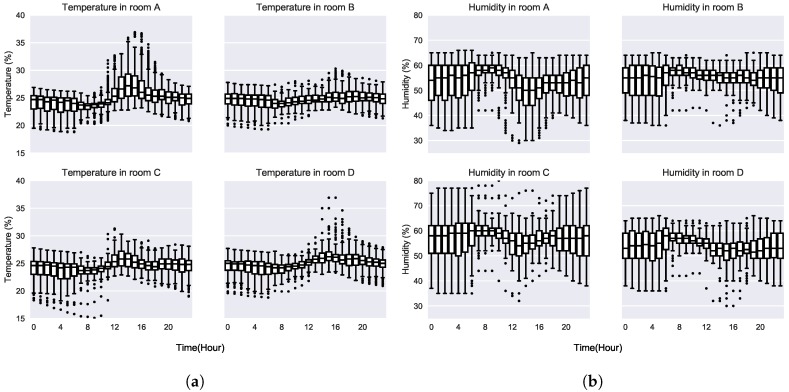
Temperature and humidity measurements in the monitored rooms: (**a**) temperature and (**b**) humidity measurements in each of the monitored rooms.

As shown in Figure 3, the temperature and humidity are maintained stable in a small variation range by the HVAC systems. It can be seen how the systems maintain the temperature and humidity values in fixed ranges, especially in the morning. Thus, the system is capable of detecting working and not working days by using this information, which could be very helpful for a plug and play device which does not require to be fed with extra information. Because temperature and humidity measurements do not provide the information needed for presence estimation the movement sensors outputs were analysed to obtain such information.

### 4.1. Occupancy of the Rooms

Passive infrared sensors only provide binary information when movement is detected and lost, because of that, they do not provide occupancy information directly making it necessary to process the data to obtain usage information. The data processing that had to be performed has been described in Section 3. After performing such modelling, data from different months were analysed obtaining information about usage of the rooms, which showed similar behaviours for each weekday (Monday-Thursday) except Friday when workers usually leave the office before.

**Figure 4:**
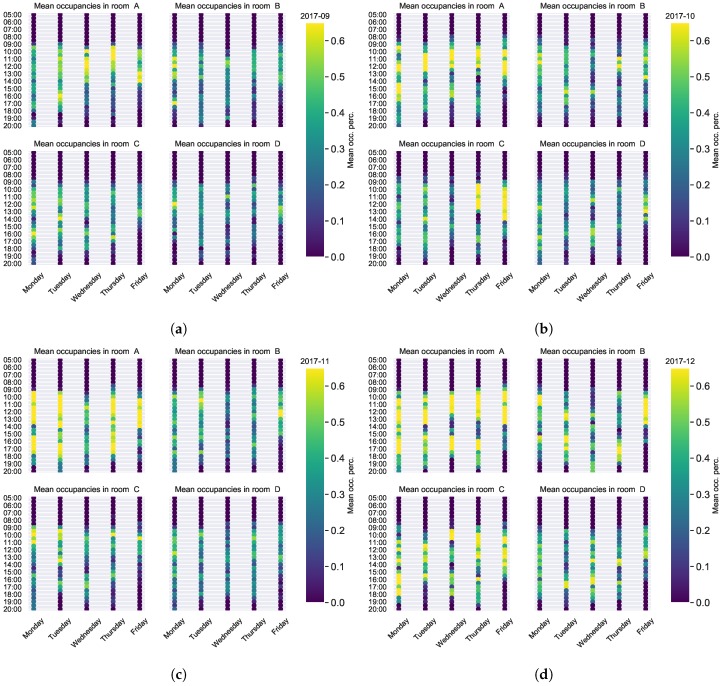
Calculated usage patterns for different months in each of the monitored meeting-rooms: (**a**–**d**) contain the usage patterns for September, October, November and December (2017) respectively.

Considering that different weekdays show similar behaviours from week to week, some usage patterns were calculated taking the average usage per half-hour intervals. Those patterns were constructed using data from individual months, showing some similar behaviours between them. To take advantage of that we propose to use those patterns as predictors for the following month. Usage patterns obtained with usage data from different months are shown in Figure 4. It can be seen how the usage from Monday to Thursday is mainly given in the morning and afternoon with a stop at midday, while on Fridays the rooms are mainly used in the morning. The mentioned patterns can clearly be seen in room A data in Figure 4d.

As it can be seen in Figure 4 the usage patterns for different months show similar behaviour where meeting-rooms are mainly used in the morning and afternoon with a break in the noon. Of course this was an expected behaviour, as it was not probable to detect usage outside working hours, but obtaining the expected results shows the validity of the proposed model. It can also be seen how the room usage on Friday afternoon is very rare. As the calculated patterns seem to be quite regular, they are used as predictors of room usage, this approach is further explained in Section 4.2.

**Figure 5:**
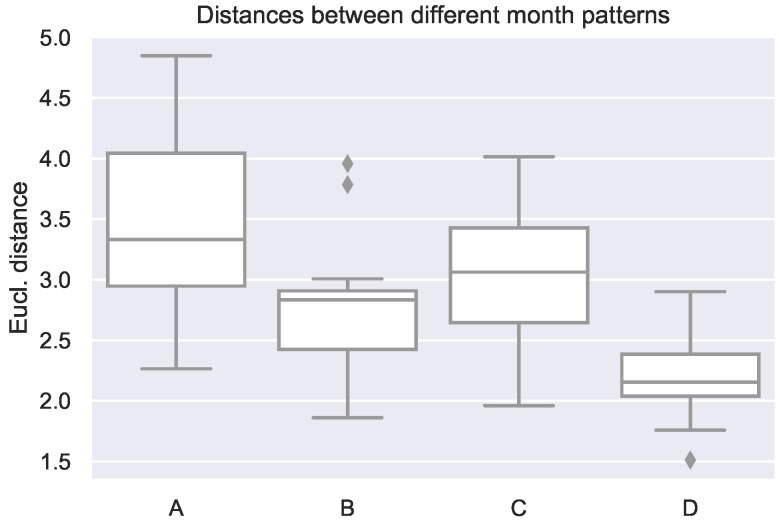
Euclidean distances between monthly usage patters for each of the monitored rooms (A–D).

When computing the dissimilarities of the patterns between different months of the same room, Euclidean distance (see Equation (1)) between them take values of the order of 1–5 when the maximum difference could theoretically be 240 (as there are 240 entries in each month pattern: 1 value per 30 min, that is 48 values a day, or 240 values a work-week (Monday to Friday)). A summary of the Euclidean distances between the different month patterns per monitored room can be seen in Figure 5. Those Euclidean distances tell that the monthly usage patterns are similar between them and in consequence that the room usage is quite regular, and therefore, it should be possible to predict it.

(1) $$d_{Eucl}\left(p,q\right)=d_{Eucl}\left(q,p\right)=\sqrt{\sum_{i=1}^{n}{\left(q_{i}-p_{i}\right)}^{2}}\;,\;n=240$$

### 4.2. Predicting Day-Ahead Room Usage

As previously shown in Figure 4, the usage patterns seem to be quite regular, which has been proved analysing the Euclidean distances of the monthly patterns, opening the way to think of them as predictors. In this first approach, we propose to use the usage patterns of one month to predict daily usage of the following month. The prediction in this case consists of using the pattern calculated for Mondays as the prediction for all Mondays of the next month. This kind of predictive methodology considers only information about previous usage, focusing on the regular usage described before.

The proposed modelling helps determining the room usage and analysing how each of the rooms is used regularly, but when it comes to feeding control techniques for systems like the HVAC the information needs to be converted to occupied/non-occupied. To obtain such information via the patterns the room is defined as occupied in an interval if the mean occupancy is at least 0.2, this way avoiding to set the room as occupied in enter-exit actions. When using such definition and applying the patterns from one month to the following month, the accuracy scores obtained are around 80%. The presented approach suffers limitations as for example not being able of updating the data with newest available data. The obtained accuracy scores are shown in Table 1. It can be seen how the months that are less accurate are August and especially September, what could be expected as are the months when most workers take their summer vacation; to consider that some other approach should be tried to consider for actual information when calculating the patterns. It can also be seen how the results are very similar in all cases showing that the room usage is quite regular.

To try to improve the predictions, another approach was implemented by calculating the predictors, that is the patterns, for weeks instead of months using patterns from the previous n weeks to calculate the actual one. That way the information used as predictor is nearer from the prediction horizon and the information updates more rapidly. The weak point of this approach is that if there is a rare event one week the prediction can be nonsense for the next week.

**Figure 6:**
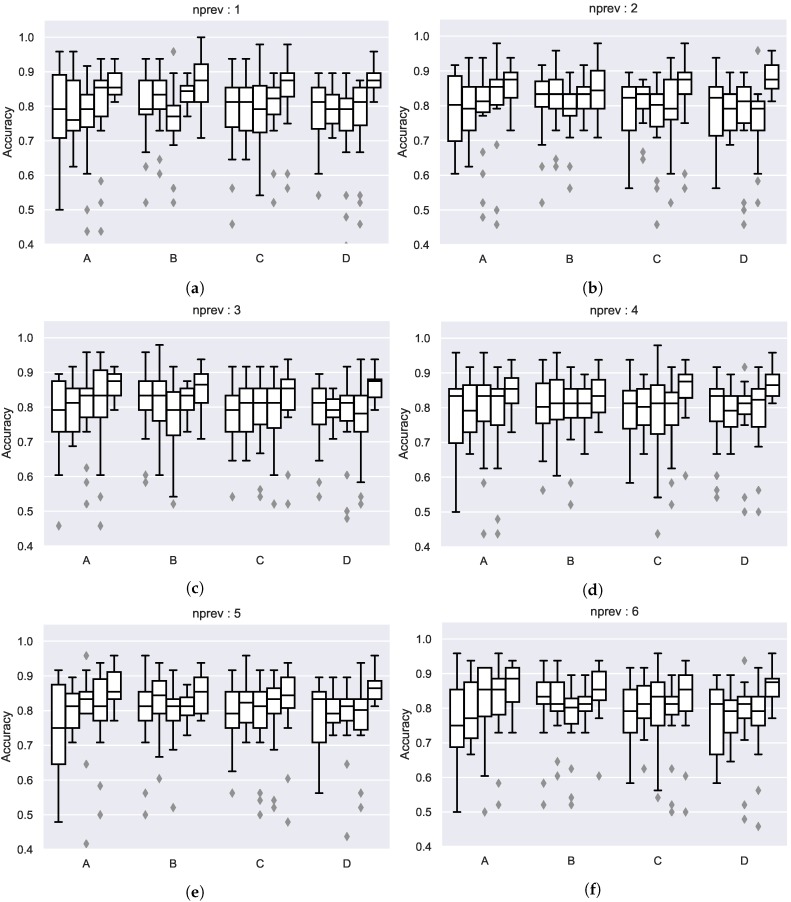
Accuracy scores of the predictions with the usage patterns using different number of previous weeks for constructing the patterns in each of the monitored meeting-rooms (separated per week-day, boxes are in order: Monday-Friday): (**a**) 1 week, (**b**) 2 weeks, (**c**) 3 weeks, (**d**) 4 weeks, (**e**) 5 weeks, (**f**) 6 weeks.

For evaluating the impact of feeding the patterns with live information, several usage patterns were calculated using different previous week configurations for each. Patterns for the previous weeks were calculated, from just using last week patterns to the last six weeks. When evaluating the patterns as predictors different accuracy scores were obtained which can be seen in Figure 6. In general, the results are similar to monthly patterns, but it can be seen how using more actual information seems to improve the results in some cases.

Results in Table 2 show that results with the proposed two approaches are similar. The mean accuracy obtained for each month, when applying patterns calculated with previous 1, 2, 3 or 4 weeks (column *Acc. prev. n weeks*) and the ones obtained when applying previous months’ patterns (column *Acc. month*) are shown. In the majority of the cases the patterns calculated once a month give better results, but there are cases, such as September, in which the mean accuracy values improve when using the n previous week approach. In this case the effect of the end of summer holidays is notorious.

The usage of the patterns as predictors shows that the results are accurate and because of their simplicity they can therefore be calculated and tested in the same device where the data acquisition is performed. That way, avoiding installing additional computing capabilities and thereby maintaining a low cost for the entire system while providing predictive capabilities.

### 4.3. Comparing Reservations and Real Usage

The installed movement sensors and the work commented on previous sections can be reused to study the correct reservation of meeting rooms, comparing the real usage with the reservations stored in the room booking system, taking advantage of its existence. In several office buildings the incorrect usage of reservations may result in having empty meeting rooms while workers are trying to reserve one without success, which in fact may lead to an important loose in productivity.

**Figure 7:**
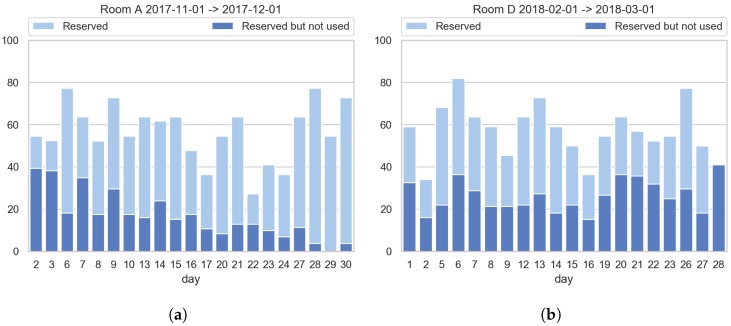
Example of good and bad usage of meeting rooms (Saturdays, Sundays and holidays have been removed): (**a**) shows a correct usage of the booking system, especially in the second half of the month. (**b**) on the contrary shows a worse usage of the booking system as a big part of the time the room is reserved but not used.

When comparing the real usage of the meeting rooms with the reservations we found that the incorrect usage of them is quite popular, with workers reserving the meeting-rooms normally around 30% more time than they really use them, even in some cases meeting rooms are reserved but they are not used. Figure 7 shows examples of correct and incorrect meeting-rooms reservation-usage relation.

**Figure 8:**
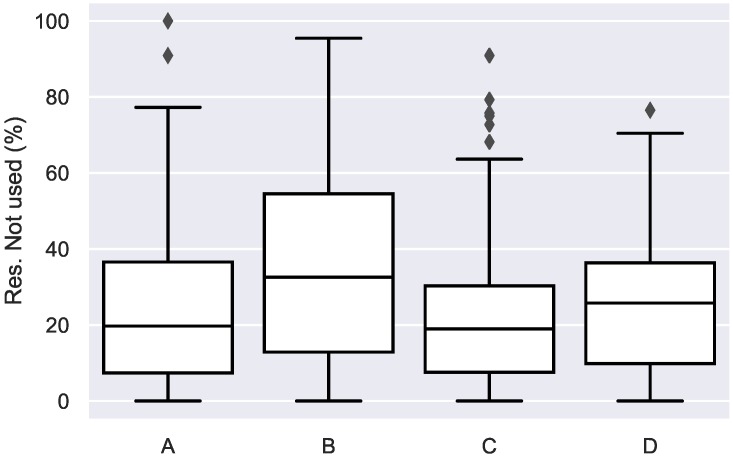
Percentages of time in which a room is reserved but not used.

When analysing the usage and reservations during the monitoring period, it can be seen that in general rooms are reserved around 25% more in mean, which is acceptable as a margin time. However, there are cases in which the rooms are reserved much more time than they should (see Figure 8). Taking that into account, some company policies should be defined to avoid those incorrect room reservations.

## 5. Conclusions

The methodology described in this paper shows some of the possibilities that a simple IoT network installation can provide. In this paper several usage patterns have been calculated, the procedure of calculating them does not require big computing resources, which makes it possible to compute them in the edge device, allowing to take the decisions in the installed infrastructure itself.

When using the patterns as predictors the accuracy scores obtained are around 80% which are more than enough for many purposes in the described application area. It has been shown how updating the patterns with the newest available information improves the results in some special cases. Considering that, an automatic system in which the patterns are updated with online information and applied for the following days seems to be a correct approach to design control scenarios for HVAC and similar devices. That way, the information obtained with the presented solution could fed Model Predictive Control strategies making it possible to adjust better human behaviour allowing to use energy more accurately.

When contrasting reservations with usage, we were able to determine how rooms are sometimes used incorrectly, reserving the rooms for much more time than they are later used. In this sense, the idea of using the installed infrastructure to determine room usage and detect reservations that are not being used should be considered. A system could be developed to cancel reservations that are not being used or at least notify the user that has made the reservation about it and make it possible to cancel the reservation if it is not going to be used.

The methodology presented in this paper has been designed to be applied in any type of room of a building, even if the study is based on measurements done in meeting-rooms. The main limitation of the proposed system is not related with the activities that can be carried out in the rooms (it just requires small movements to activate the sensors), but by the detection range of PIR sensors. The system cannot guarantee the correct functioning in rooms bigger than the detection range of the used PIR sensors, this can be fixed for those cases by a minor upgrade consisting of deploying more PIR sensors distributed through the room covering the entire space and defining occupancy as the output of a logical OR gate applied to all the PIR outputs.

The proposed system and methodology is also interesting to consider for domestic monitoring. In this particular case the nature of the patterns will vary depending on the type of user. The expected pattern will be totally different in the case of a worker who is outside home for most part of the day, or in the case of an elderly person who spends most part of the day at home. In the domestic case we expect the patterns to be more stable and regular as the number of users is sensibly smaller, that way reducing the variance. Such nature opens the possibility to detect not only the usage of the different spaces of the house, but to detect behaviours such as the regular breakfast hour, or detecting when a person returns home from work, allowing to have a smarter monitoring.

In the future, we intend to research deeper in the reservation information to be able to predict room usage information from previous knowledge and the registered room bookings for the following days. We intend to check if this complementary information can help to improve the predictions capabilities described in this paper, as well as testing the proposed configuration in this paper in other scenarios, especially in houses.

## Figures and Tables

**Table 1:** Accuracy scores obtained applying patterns from month (t − 1) to month t.

Month	Pattern from Month	Accuracy
8	7	0.81
9	8	0.78
10	9	0.83
11	10	0.82
12	11	0.82

**Table 2:** Means of the accuracy scores obtained applying patterns calculated with the previous n week data, and patterns obtained monthly.

Month	n	Acc. Prev. n Weeks	Acc. Month
8	1	0.81	0.81
8	2	0.78	0.81
8	3	0.78	0.81
8	4	0.79	0.81
9	1	0.79	0.78
9	2	0.80	0.78
9	3	0.80	0.78
9	4	0.80	0.78
10	1	0.80	0.83
10	2	0.81	0.83
10	3	0.81	0.83
10	4	0.79	0.83
11	1	0.81	0.82
11	2	0.81	0.82
11	3	0.81	0.82
11	4	0.81	0.82
12	1	0.81	0.82
12	2	0.81	0.82
12	3	0.81	0.82
12	4	0.81	0.82

## Abbreviations

The following abbreviations are used in this manuscript:

IoT	Internet of Things
MPC	Model Predictive Control
HVAC	Heating, Ventilating and Air-Conditioning
SMR	Smart Meeting Room
HEMS	Home Energy Management System
PIR	Passive Infrared Sensor
CRE	Corporate Real Estate
